# Investigation of annular pancreas through multiple detector spiral CT (MDCT) and MRI

**DOI:** 10.1002/acm2.13487

**Published:** 2021-11-29

**Authors:** Yongxia Zhou, Xiaoyan Li

**Affiliations:** ^1^ The Yongchuan Affiliated Hospital Chongqing Medical University Chongqing China

**Keywords:** annular pancreas, computed tomography, magnetic resonance cholangiopancreatography, magnetic resonance imaging, retrospective study

## Abstract

**Background and purpose:**

To investigate annular pancreas in adults using imaging features displayed on computed tomography (CT) and magnetic resonance imaging (MRI).

**Methods:**

Retrospective review of annular pancreas in patients undergoing CT or MRI examinations. Two abdominal radiologists blindly reviewed the CT, MRI, and magnetic resonance cholangiopancreatography (MRCP) images from the Picture Archiving and Communication Systems (PACS). A Kruskal–Wallis test was performed to evaluate subjective scoring, with Mann–Whitney test for the comparison. A *p*‐value less than 0.05 was considered statistically significant.

**Results:**

Eleven patients (45.8%) presented a complete ring of pancreatic tissue surrounding duodenum, displayed as circular, triangular, or sandwich sign configuration, the other 13 patients (54.2%) had incomplete annular pancreas which displayed a crocodile jaw appearance, pancreatic tissue extending in a posterolateral or anterolateral direction towards duodenum. When comparing CT images of each sequence, the arterial phase group had the highest score compared with the venous phase and the plain film group (*χ* ^2^ = 58.21, *p* < 0.05). When comparing MRI enhancement volumetric interpolated breath‐hold examination (VIBE) sequences, arterial phase group scores were the highest (*χ* ^2^ = 18.98, *p* < 0.05).

**Conclusions:**

Both CT and MRI could detect annular pancreas, with artery phase being the best sequence to diagnose annular pancreas.

## 1 INTRODUCTION

Annular pancreas is a rare congenital anomaly where the enlarged head of the pancreas completely or partially encircles the duodenum, usually diagnosed in neonates after suffering from varying degrees of gastric outlet obstruction.^1^, ^2^ Annular pancreas is uncommon in adults as it is usually asymptomatic and often detected incidentally.^3^, ^4^, ^5^ Common symptoms include abdominal pain, excessive flatus, peptic ulcers, vomiting, pancreatitis, or biliary obstruction.^6^, ^7^, ^8^ Annular pancreas can be easily misdiagnosed as a pancreatic head or duodenal tumor due to insufficient understanding of this congenital anomaly, which can result in unnecessary surgery.

The gold standard for the diagnosis of annular pancreas in adults is endoscopic retrograde cholangiopancreatography (ERCP) or laparotomy.^9^ However, these procedures are invasive. Due to advances in diagnostic imaging technology and increased understanding of this anatomic variability, multiple detector spiral computed tomography (MDCT), magnetic resonance imaging (MRI), and magnetic resonance cholangiopancreatography (MRCP) have made noninvasive diagnosis of annular pancreas a reality. A literature review has revealed that annular pancreas has scarcely been reported, with few large sample systematic studies.^10^ In order to improve understanding of this disease and provide help for clinics, we retrospectively analyzed the clinical and imaging data of 24 cases of annular pancreas, investigated the imaging features of annular pancreas in adults, and found the best sequence for diagnosis of annular pancreas.

## 2 METHODS

### 2.1 Patients

A review of computed tomography (CT) and MRI databases from our institution between January 2011 and November 2019 revealed 24 patients with annular pancreas within the Picture Archiving and Communication Systems (PACS). All cases of radiologically incomplete annular pancreas were diagnosed by laparotomy or ERCP.^11^ This retrospective study was approved by the Institutional Review Board of Yongchuan Hospital of Chongqing Medical University with a waiver of the requirement for informed consent.

### 2.2 Imaging techniques

#### 2.2.1 Computed tomography

CT studies were performed with 8‐ or 256‐MDCT scanner. Parameters utilized on the GE LightSpeed Ultra scans were as follows: reference tube voltage and tube current: 120 kV and 200 mA; pitch: 1.000; layer and reconstruction layer thickness: 2 mm; 512 × 512 matrix. Philips Brilliance iCT scanner parameters were reference tube voltage: 120 kV with automatic tube current time; pitch: 0.899; layer and reconstruction layer thickness: 2 mm; 512 × 512 matrix. Patients lying supine and holding their breath were scanned from the diaphragmatic dome to the ala of the ilium. Note that 80 ml of iohexol (370 mgI/ml) at 5 ml/s were administered intravenously (IV) for all CT examinations after a plain scan, followed by 15 ml of normal saline. After injecting contrast agent for 30 or 65 s, either an artery or venous phase scan was performed, respectively.

#### 2.2.2 Magnetic resonance imaging

Magnetic resonance (MR) images were obtained using a Magnetom Verio 3.0‐T scanner using an array body coil. Scans were performed from the diaphragmatic dome to the inferior border of the liver with patients lying supine and holding their breath. The protocol included: axial T1WI‐Fast Low Angle SHot (T1WI‐FLASH), axial T2WI half‐Fourier acquisition single‐shot turbo spin‐echo (T2WI‐HASTE), coronal true fast imaging with steady‐state precession (True‐FISP), and 3D MRCP imaging, obtained with high‐resolution fast spin‐echo and a maximum intensity projection technique. Multiphase‐dynamic contrast‐enhanced MRI was performed using a volumetric interpolated breath‐hold examination (VIBE) sequence. After injection of gadopentetate dimeglumine (Gd‐DTPA, 0.2 mmol/kg, 2–3 ml/s) over 20–25 s, arterial phase images were obtained, portal venous phase images after 55–65 s, with parenchymal phase imaging performed after 90–120 s. Equilibrium‐phase T1WI imaging was performed after the VIBE sequence.

### 2.3 Interpretation

Electronic clinical data of the patients subject to review were obtained, including symptoms, surgical approach, and pathologic results. Two abdominal radiologists blindly reviewed the CT, MRI, and MRCP images from the PACS. The final diagnosis was reached by the two radiologists after analysis of the images and discussion. The diagnostic criteria of annular pancreas were: the pancreas had completely or incompletely encircled the duodenum in the CT or MRI imaging, or MRCP showed an annular pancreatic duct.^12^

For each patient the following findings were established: (i) imaging features and complications – the type and shape of annular pancreas, the section and degree of annular pancreatic tissue encircling the duodenum, and observation of the annular pancreatic duct, pancreatic division, and other complications. (ii) Duodenal wall and duodenal cavity using a five‐point scale (5: excellent tissue contrast; 4: good tissue contrast; 3: moderate tissue contrast; 2: hazy tissue contrast; 1: evaluation not possible) to evaluate image quality for annular pancreas particularly tissue contrast differentiating pancreatic tissue.

### 2.4 Statistical analysis

Statistical analysis was performed using SPSS version 19.0. A Kruskal–Wallis test was performed to evaluate subjective scoring, with Mann‐Whitney test for comparison of two groups. The Mann–Whitney test was used for comparing the scoring of T1WI and T2WI. The degree of agreement between two radiologists in the grading of image quality was calculated using the Kappa statistic (*k*) where the results were interpreted as poor (*k* ≤ 0.20), fair (*k* > 0.20–0.40), moderate (*k* > 0.40–0.60), good (*k* > 0.60–0.80), or excellent (*k* > 0.80). A *p*‐value less than 0.05 was considered statistically significant.

## 3 RESULTS

### 3.1 Clinical characteristics of annular pancreas

This study included a total of 14 females and 10 males, with a median age at the time of diagnosis of 47 years (ranging from 18 to 76 years). The clinical features were abdominal pain in 15 cases, cholecystolithiasis associated with pancreatic neoplasm in one case and abdominal distention in one case (Table 1). The finding of annular pancreas was incidental when undergoing imaging examination for cancer staging or other diseases in the remaining seven cases. Among all patients, 12 cases (50%) had suffered from or had a history of biliary calculi.

**TABLE 1:** The clinical characteristics of annular pancreas

Clinical symptoms	No.	Comorbidities
Abdominal pain (15)	5	Cholecystolithiasis
	3	Cholecystolithiasis and cholecystectomy
	1	Left intrahepatic bile duct calculi
	1	Cholecystolithiasis and acute pancreatitis
	1	Acute pancreatitis
	1	Cholecystolithiasis and superior mesenteric artery syndrome
	2	Gastrointestinal ulcer
	1	Abdominal pain
Paroxysmal vertigo and hand tremors (1)	1	Cholecystolithiasis
Abdominal distention (1)	1	Liver cirrhosis
Cancer (4)	1	Esophagus cancer
	1	Lymphoma
	1	Rectal cancer
	1	Breast cancer
Others (3)	3	NA^a^

^a^ Two were found by physical examination; one had a poor appetite and emaciation for 3 months.

### 3.2 CT, MRI, and MRCP findings

**FIGURE 1:**
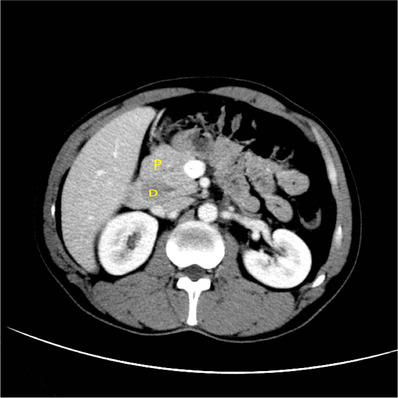
A 39‐year‐old man with abdominal pain. Axial multiple detector spiral computed tomography (MDCT) venous phase shows a triangular configuration of complete annular pancreas. P, pancreas; D, duodenum

**FIGURE 2:**
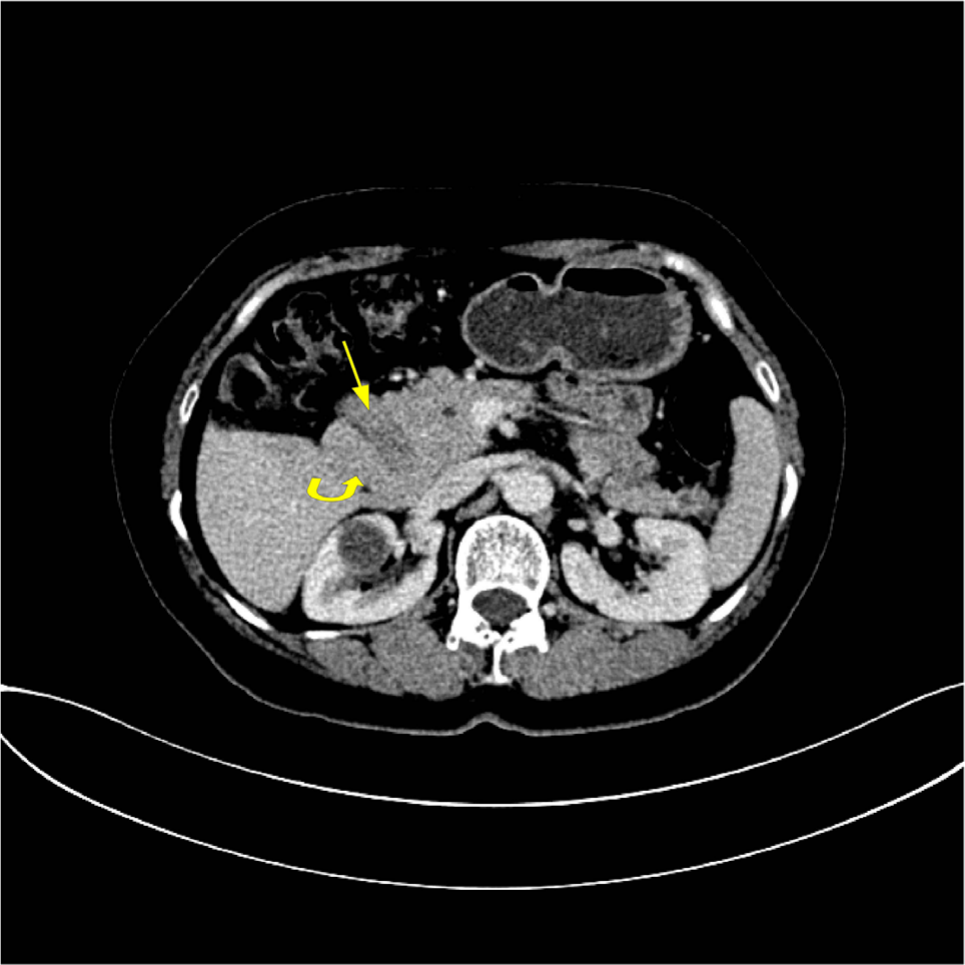
A 50‐year‐old woman with abdominal pain. Axial multiple detector spiral computed tomography (MDCT) venous phase shows duodenum (arrow) completely surrounded by the head of the pancreas (curved arrow), a sandwich sign appearance

**FIGURE 3:**
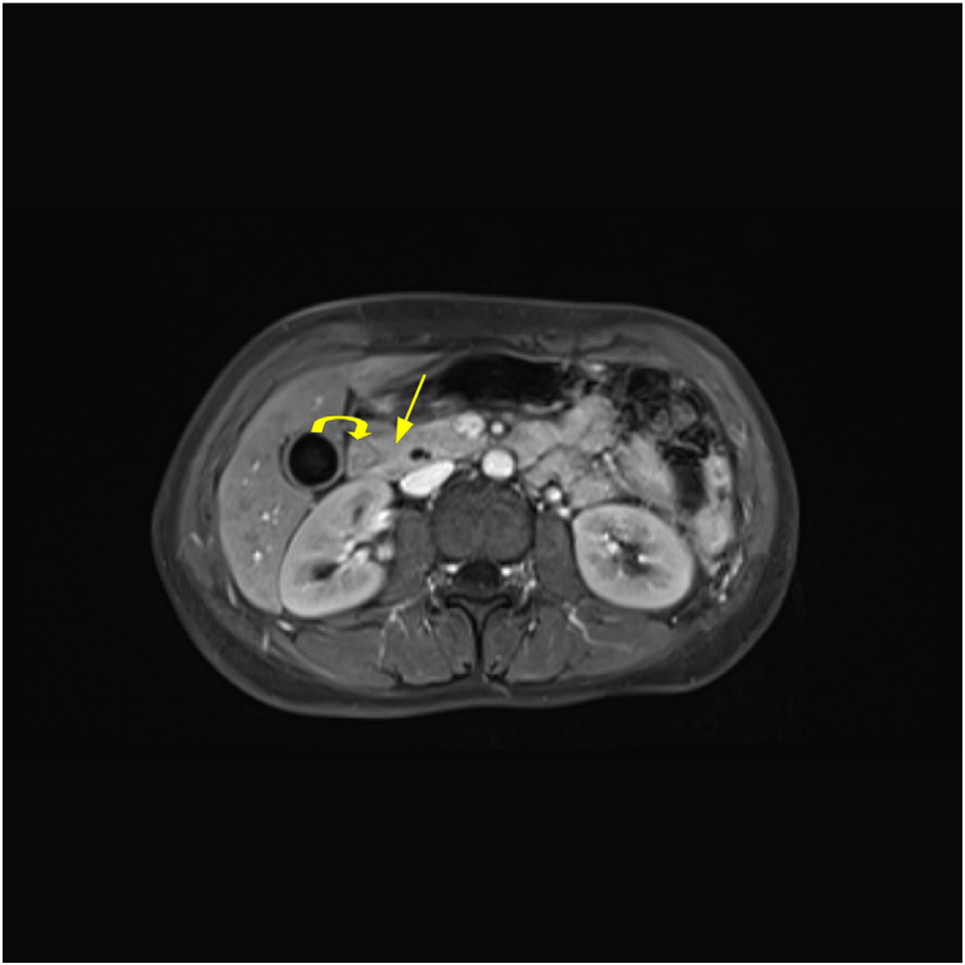
A 24‐year‐old woman with intermittent episodes of abdominal pain and vomiting. Axial fat suppressed contrast‐enhanced T1‐volumetric interpolated breath‐hold examination (VIBE) arterial phase which shows the duodenum (curved arrow) is partially encircled by the head of pancreas (arrow), a crocodile jaw appearance

**FIGURE 4:**
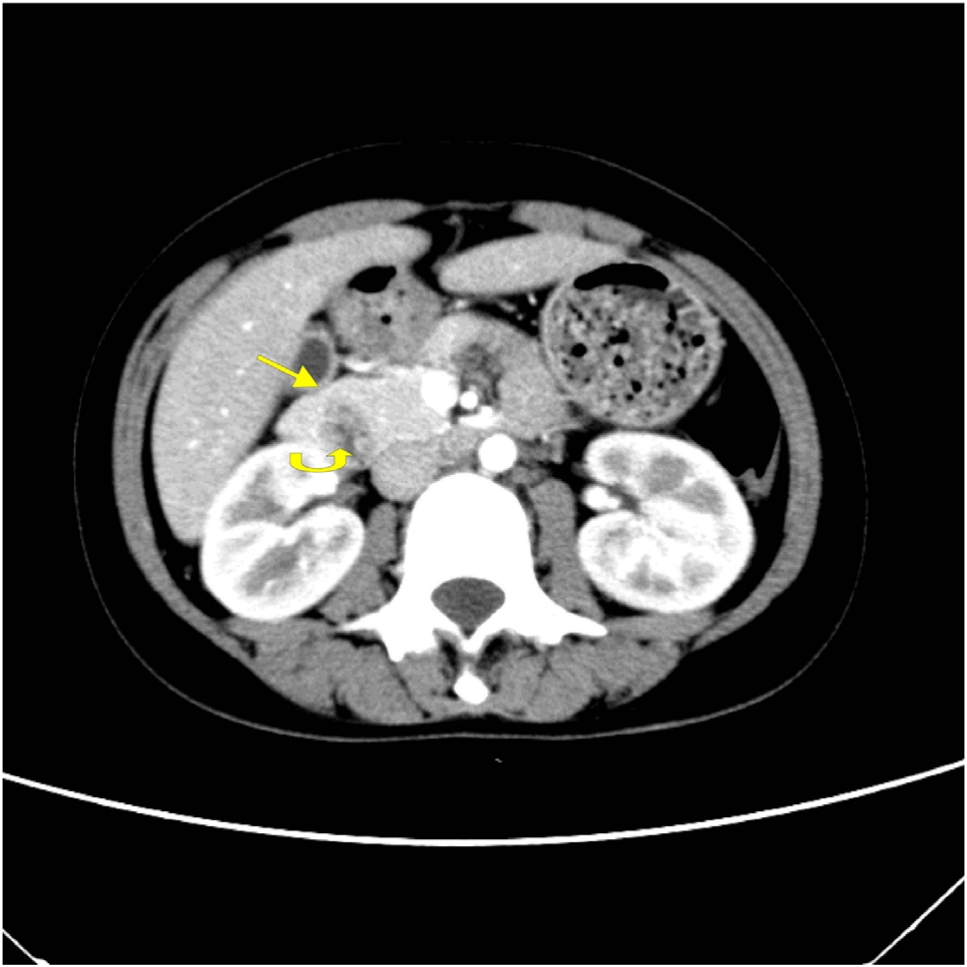
A 22‐year‐old woman with abdominal pain. Axial multiple detector spiral computed tomography (MDCT) venous phase shows pancreatic tissue (arrow) extending in an anterolateral direction towards the duodenum (curved arrow)

**FIGURE 5:**
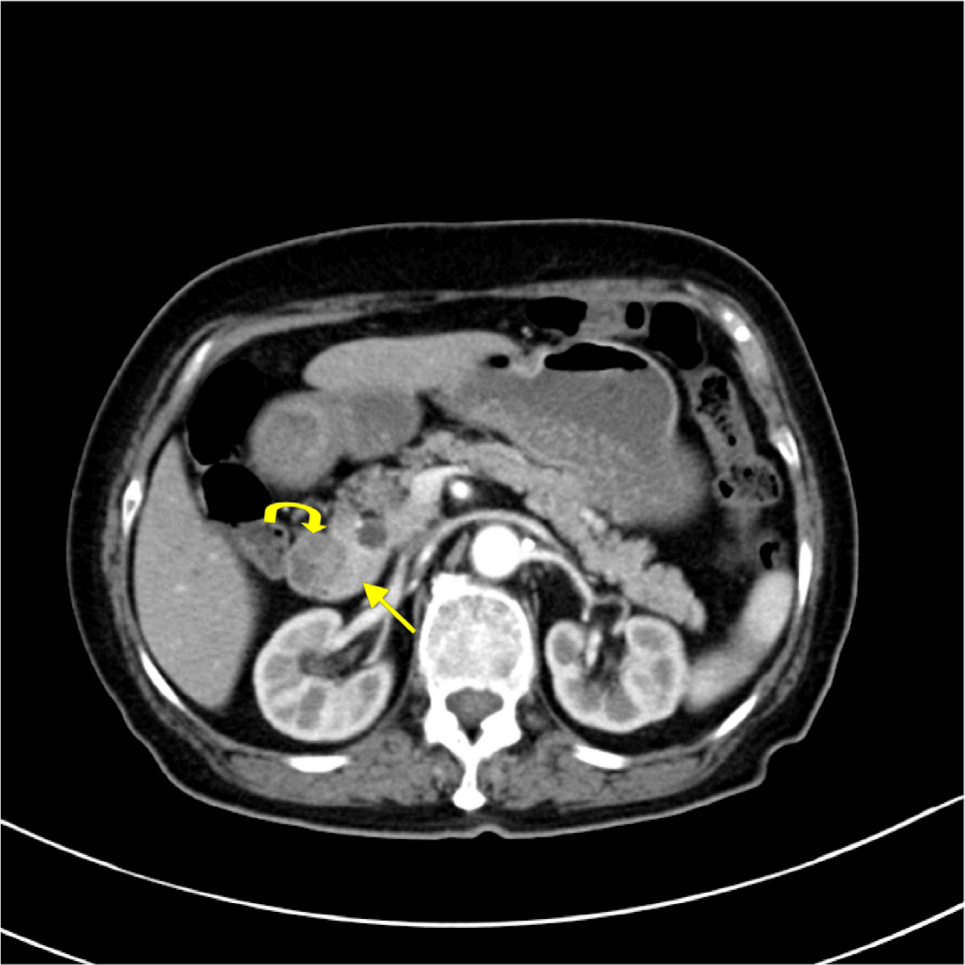
A 71‐year‐old woman with esophageal carcinoma. Axial multiple detector spiral computed tomography (MDCT) arterial phase shows pancreatic tissue (arrow) extending in a posterolateral direction towards the duodenum (curved arrow)

**FIGURE 6:**
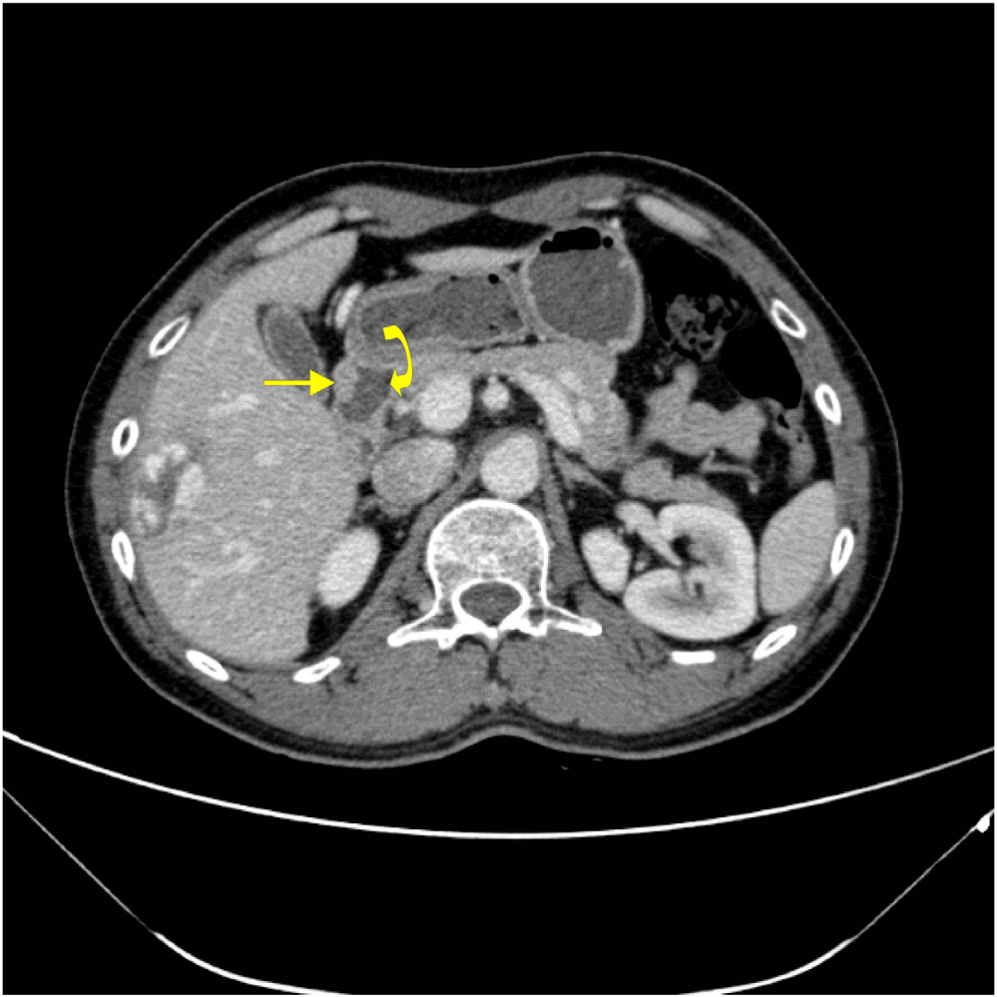
A 44‐year‐old man with hepatic carcinoma. Axial multiple detector spiral computed tomography (MDCT) arterial phase shows pancreatic tissue (arrow) surrounding the posterior wall of the duodena bulb (curved arrow)

**FIGURE 7:**
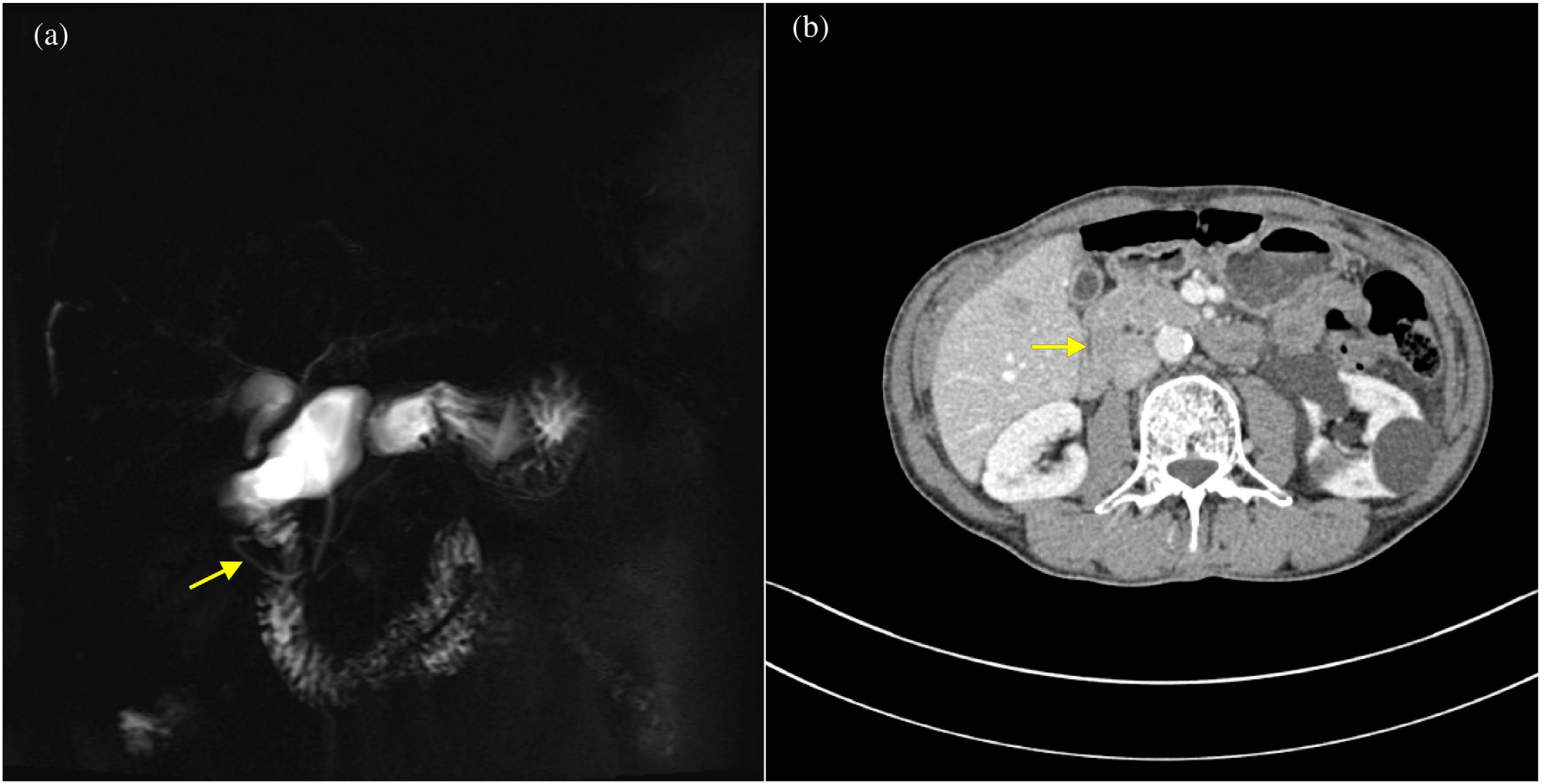
Magnetic resonance cholangiopancreatography (MRCP) image (a) shows the whole annular duct (arrow). Axial multiple detector spiral computed tomography (MDCT) (b) partially displays the annular duct in different slices of computed tomography (CT) imaging

**FIGURE 8:**
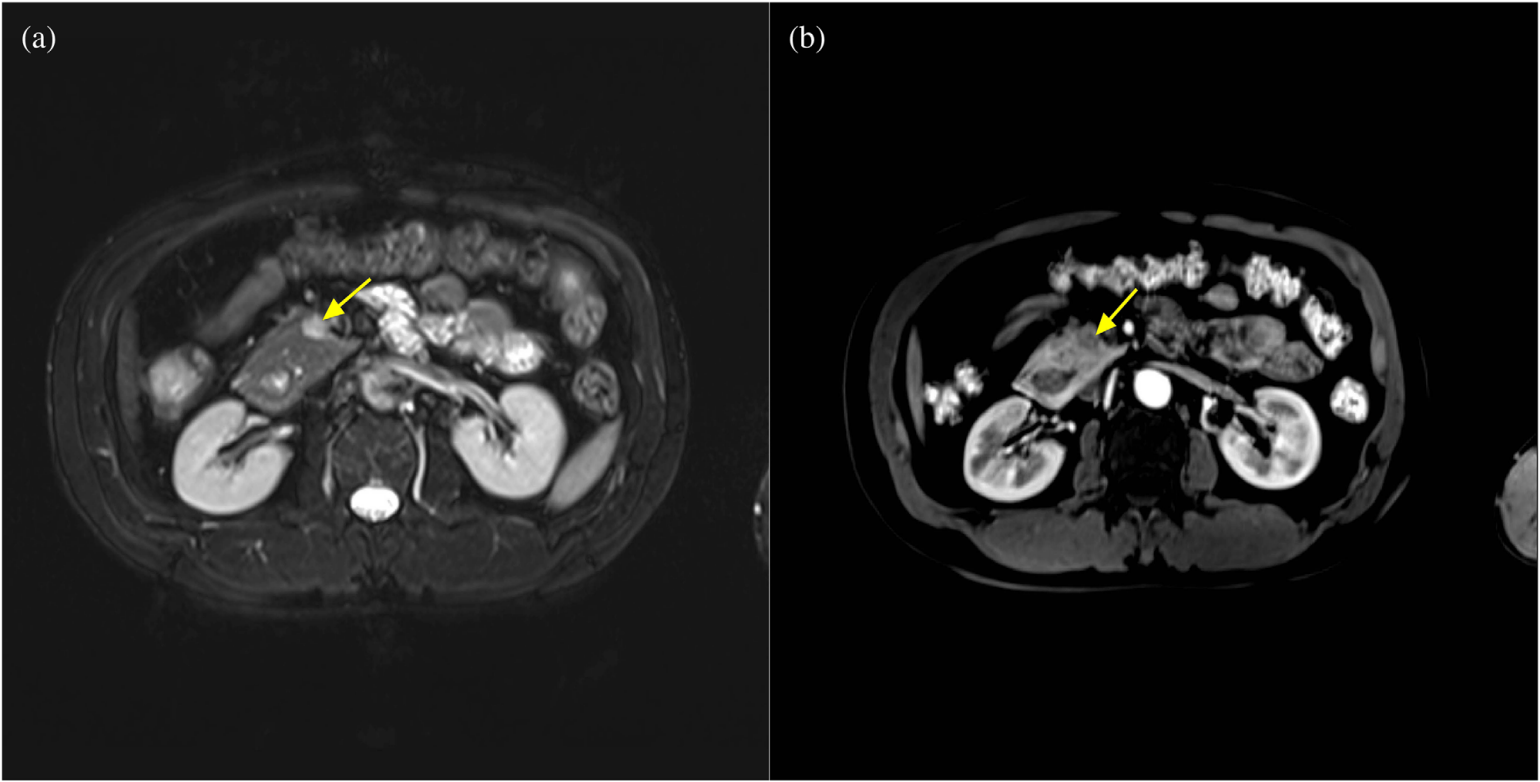
A 45‐year‐old man with annular pancreas and insulinoma. (a) Axial fat suppressed T2‐weighted magnetic resonance (MR) image shows neoplasm (arrow) in the pancreatic head which manifests as hyperintensity. (b) Axial fat suppressed T1‐volumetric interpolated breath‐hold examination (VIBE) arterial phase MR image shows that the neoplasm is slightly enhanced

Of these, 23 patients received dynamic contrast enhancement CT scans, eight received MRCP and gadolinium contrast enhancement MRI, seven received CT, MRI, and MRCP, with only one patient receiving MRI and MRCP. The key parameters of the MR protocol are shown in Table 2. The fat space between the pancreatic head and duodenum was not observed in any of the 24 cases. Eleven patients (45.8%) had a complete ring of pancreatic tissue surrounding the duodenum which displayed circular, triangular (Figure 1), or a sandwich sign configuration (Figure 2), indicating complete annular pancreas. The other 13 patients (54.2%) presented with an incomplete annular pancreas which displayed a crocodile jaw appearance (Figure 3), pancreatic tissue extending in an anterolateral (Figure 4), or posterolateral direction (Figure 5) to the duodenum, indicating an incomplete pancreas. The configurations of annular pancreas are summarized in Table 3. In the 13 cases of incomplete pancreas, the perimeter of pancreatic tissue encircling the duodenum could be characterized as follows: one half circumference in three cases, more than a half to three quarters in eight cases, and more than three quarters in two cases. We observed two modalities of pancreatic tissue surrounding the duodenum: surrounded the descending portion of the duodenum occurred in 22 cases (91.7%), while surrounded the posterior wall of the duodenal bulb in two incomplete cases (8.3%) (Figure 6), which were established during surgical exploration. An annular pancreatic duct was found in seven cases using both CT and MRI. Moreover, MRCP was superior in showing the whole annular pancreatic duct (Figure 7). Eight patients also had cholecystolithiasis and one patient had a hepatolith in the left hepatic duct. There were five cases with a dilated biliary system and main pancreatic duct, of which three had received a cholecystectomy due to cholecystolithiasis. Another two cases were associated with cholecystolithiasis and distal choledochus obstruction. The remaining cases were associated with other complications, including pancreatic division in six cases (25%), pancreatitis in two cases (8.3%), superior mesenteric artery syndrome in one case, and pancreatic neoplasm (insulinoma established by postoperative pathology) in one case (Figure 8).

**TABLE 2:** The key parameters of the magnetic resonance (MR) protocol

	TR (ms)	TE (ms)	Slice thickness (mm)	Slice gap (mm)	Matrix	FOV (mm^2^)
HASTE coronal T2WI	800	90	4	0.8	256 × 256	400 × 400
Transverse diaphragm navigation FS FSE T2WI	2300	105	6	1.8	320 × 298	400 × 400
OPP‐IN FLASH Transverse	120	TE1: 1.23 TE2: 2.46	6	1.8	256 × 256	400 × 325
HASTE coronal multi‐angle rotation	4500	741	50	25	384 × 269	300 × 300
3D MRCP T2 SPACE	2000	703	1.4	/	384 × 384	380 × 380
VIBE	4.15	2.01	3	0.6	320 × 240	400 × 400

Abbreviations: FOV, field of view; FS, fat suppression; FSE, fast spin‐echo; MRCP, magnetic resonance cholangiopancreatography; OPP, out‐of‐phase; SPACE, sampling perfection with application optimized contrast using different flip angle evolution; TE, echo time; TR, repetition time; VIBE, volumetric interpolated breath‐hold examination.

**TABLE 3:** Configuration of annular pancreas

	Complete annular pancreas (*n* = 11)			Incomplete annular pancreas (*n* = 13)		
Configuration	Circular	Triangular	Sandwich sign	Crocodile jaw	Anterolateral	Posterolateral
Number	4	5	2	5	3	5

**FIGURE 9:**
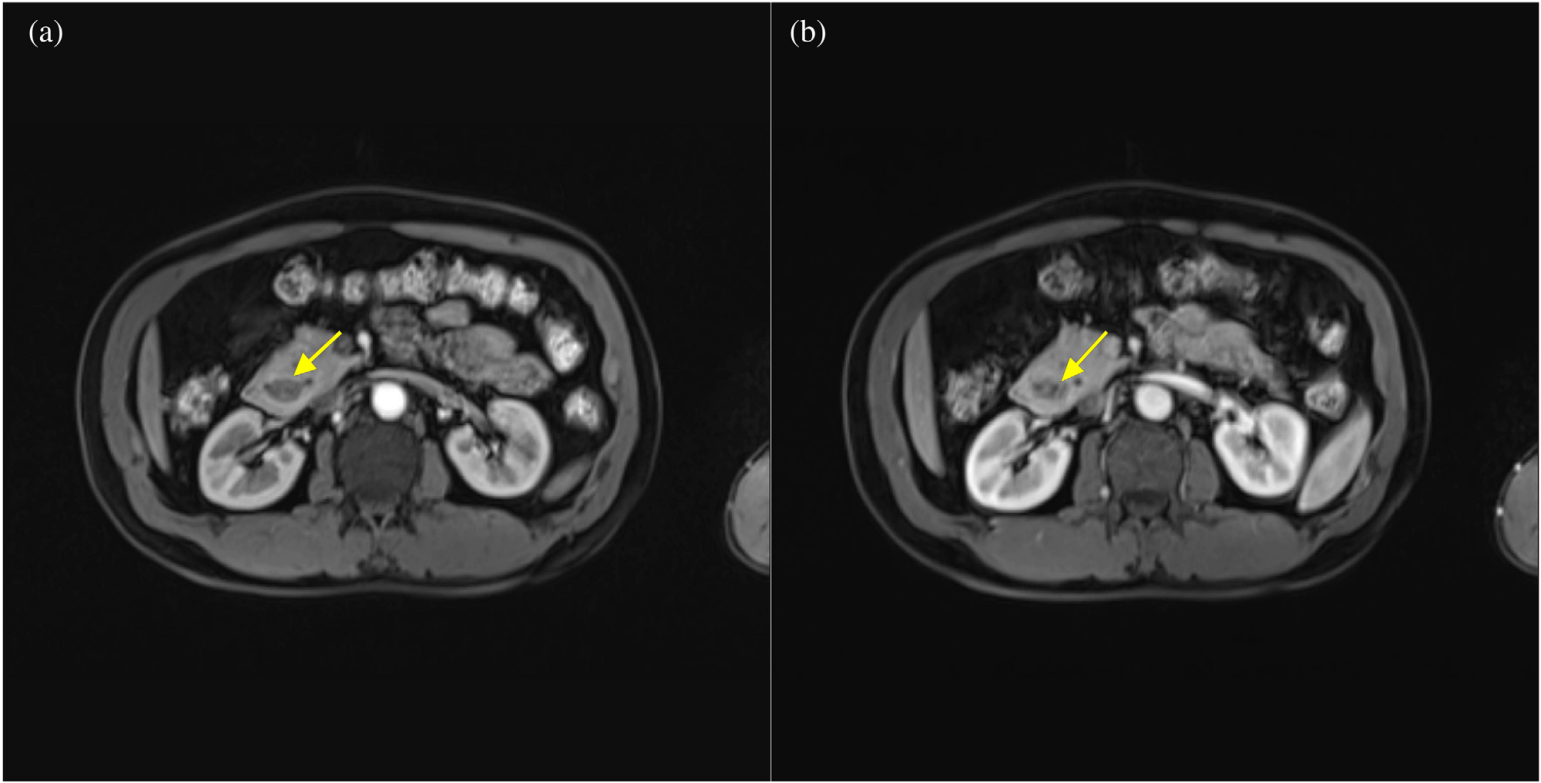
The same patient as shown in Figure 8. Axial fat suppressed contrast‐enhanced T1‐volumetric interpolated breath‐hold examination (VIBE) arterial phase (a) and venous phase (b) magnetic resonance (MR) images showing that the arterial phase is superior to the venous phase in displaying an annular pancreas. The duodenum (arrows) is completely encircled by the pancreatic head, indicating a complete annular pancreas

Both CT and MRI could detect annular pancreatic tissue, with scores of the arterial phase higher than the other sequences. For the small number of patients that underwent MRI with a different slice scanning thickness, comparison of the CT and MRI images was not performed. There was good consistency between the two independent observers' measurements (Kappa = 0.59∼1, *p *< 0.05). The scores are summarized in Table 4. When comparing the scores of the CT scans, the arterial phase group were highest, compared with the venous phase and plain scan groups, with statistically significant difference (*χ* ^2^*^^* = 58.21, *p *< 0.05), as well as between each two groups of CT scan (*p *< 0.05). The scores of T1WI were higher than T2WI, although not statistically different (*U* = 15.50, *p *= 0.07). When comparing the scores of the enhanced MRI VIBE sequence images, the arterial phase group was the highest, while the portal phases group was strongly higher than the venous group (*χ^2^* = 18.98, *p *< 0.05; Figure 9). Furthermore, there were also significant differences between each two groups of MRI sequence (*p *< 0.05).

**TABLE 4:** Comparison of annular pancreas scores of computed tomography (CT) and magnetic resonance imaging (MRI) images

	CT			MRI				
Scanning mode	Plain scan	Artery phase	Venous phase	T1WI	T2WI	Artery phase	Portal phase	Venous phase
Median	3	5	4	4	3	5	4	3
Lower quartile	2	5	4	4	2.25	5	4	3
Upper quartile	3	5	4	5	4.75	5	4	4
*χ* ^2^/*U*	49.08			15.50		18.98		
*p*	<0.05			0.07		<0.05		

## 4 DISCUSSION

The study indicated that both MDCT and MRI could display annular pancreatic tissue well, particularly contrast enhancement arterial phase, which was highly recommended for the diagnose of annular pancreas. In addition, incomplete annular pancreas was strongly suggested when the fat space between the pancreas and duodenum disappeared and the perimeter of pancreatic tissue encircled the duodenum wall by more than a half.

Approximately one in every 20 000 newborns is affected by annular pancreas^7^, ^13^ and most are associated with other malformations. However, the incidence in adults varies from 0.005% to 0.015% with no difference between the sexes.^7^, ^14^ Zyromski et al.^6^ reviewed 103 cases indicating that annular pancreas was as common in adults as in children. Due to the low incidence of annular pancreas and insufficient understanding of this disease, it is easy to misdiagnosis or miss a diagnosis. From the embryologic development of the pancreas, it is suggested that the pancreas develops from a single dorsal and two ventral buds. The two ventral buds unite and rotate with the duodenum to form the uncinate process and head of the pancreas, with the dorsal bud forming the body and tail.^3^ Several hypotheses have been proposed to explain the embryogenesis of annular pancreas, but no consensus has yet been reached.^12^

In adults with annular pancreas, two thirds are asymptomatic for life, and the mains present with pancreatitis.^11^ However, in our study, only two cases (8.3%) suffered from pancreatitis, with the most common complications being biliary calculi (50%) and biliary system obstruction (20.8%), similar to observations reported by Tewari et al.^15^

Moon^16^ believed that annular pancreas couldn't present in the first or third part of the duodenum. However, there were two cases of incomplete annular pancreas surrounding the posterior wall of duodenal bulb in our study. Furthermore, Kobayashi et al.^17^ reported a case of a ventral pancreas encompassing the pyloric ring. Sandrasegaran et al. ^11^ reported on the morphologic features of annular pancreas were either a triangular and crocodile jaw appearance, which suggested the presence of incomplete annular pancreas. In our study, the morphology of a complete annular pancreas was different from that of an incomplete annular pancreas. The complete annular pancreas was circular, triangular, or sandwich sign, while, an incomplete annular pancreas had the appearance of a crocodile jaw, in either a posterolateral or anterolateral direction of pancreatic tissue extending to the duodenum. Our research indicated that an incomplete annular pancreas was strongly suggested when the fat space between the pancreas and duodenum disappeared and the perimeter of the pancreatic tissue encircled the duodenum wall by more than half.

MRCP can show an annular pancreatic duct or pancreas divisum. In our cohort, although annular pancreatic duct could be observed using both CT and MRI in different segments, MRCP was able to show a whole annular pancreatic duct with the duodenum in the middle. The coexistent of annular pancreas with a pancreas divisum was found in 25% patients in our study, far beyond the prevalence occurred in 4%–15% of autopsy specimens and in 2%–8% of ERCP findings.^18^, ^19^, ^20^ The incidence (four in 24) of annular pancreas accompanied with tumors was substantial in our study, particularly that associated with insulinoma in the pancreatic head. However, no reports revealed the association between annular pancreas and a tumor.

Zyromski et al.^6^ and Wani et al.^21^ considered that diagnosis of annular pancreas might require surgical confirmation in more than 40% cases even in this era of radiological sophistication. They thought CT was limited by the narrowness of the ring and by the fact that the pancreatic ring lied intramurally in the duodenum without space in between. However, in our study, MDCT and MRI could both display annular pancreatic tissue well. And MRI using contrast enhancement scan and VIBE sequences was helpful for displaying annular pancreas, due to the contrast agent increasing the density or the signal intensity especially in the arterial phase. Therefore, careful observation CT or MRI arterial phase (excluding any disturbance of the blood circulation) was recommended for highly suspected cases.

A limitation of our study was that this was a retrospective investigation, which couldn't show the prevalence of annular pancreas. Thus, the aforementioned selection bias could not be neglected. Evidence with prospective data to further evaluate the value of CT or MRI for the diagnosis of annular pancreas invasively is needed. In addition, the accuracy and robustness of integrating imaging features (CT or MRI) of annular pancreas into the existing workflow is warranted to be further explored in the future.

The prevalence of annular pancreas in adults is low and these patients tend to present concurrently with biliary calculi. Misdiagnosis or missed diagnosed easily occurs in incomplete annular pancreas. Both MDCT and MRI can display annular pancreatic tissue well, particularly contrast enhancement arterial phase, which is highly recommended to diagnose annular pancreas.

## CONFLICT OF INTEREST

The authors declare no conflict of interest.

## ETHICAL APPROVAL

All procedures performed in studies involving human participants were in accordance with the ethical standards of the institutional and/or national research committee and with the 1964 Helsinki declaration and its later amendments or comparable ethical standards.

## AUTHOR CONTRIBUTIONS

Yongxia Zhou was dedicated to the integrity of the entire study, study design, definition of intellectual content, literature research, experimental studies, data acquisition, data analysis and statistical analysis, manuscript editing and manuscript review. Xiaoyan Li focused on the study concepts and design, definition of intellectual content, clinical and experimental studies, data acquisition and analysis, statistical analysis, manuscript preparation and editing. All authors have read and approved this article.

## FUNDING

This study was supported by the grant from the Chongqing city health and Family Planning Commission of China (grant numbers 2016MSXM053).

## Data Availability

The datasets used or analyzed during the current study are available from the corresponding author on reasonable request.
